# Superoxide drives progression of Parkin/PINK1-dependent mitophagy following translocation of Parkin to mitochondria

**DOI:** 10.1038/cddis.2017.463

**Published:** 2017-10-12

**Authors:** Bin Xiao, Xiao Deng, Grace G Y Lim, Shaoping Xie, Zhi Dong Zhou, Kah-Leong Lim, Eng-King Tan

**Affiliations:** 1 Department of Neurology, National Neuroscience Institute, Singapore; 2 Neurodegeneration Research Laboratory, National Neuroscience Institute, Singapore; 3 Department of Research, National Neuroscience Institute, Singapore; 4 Department of Physiology, National University of Singapore, Singapore; 5 Department of Neurology, Singapore General Hospital, Singapore; 6 Duke-NUS Medical School, National University of Singapore,Singapore

## Abstract

Reactive oxygen species (ROS) and mitophagy are profoundly implicated in the pathogenesis of neurodegenerative diseases, such as Parkinson’s disease (PD). Several studies have suggested that ROS are not involved in mitochondrial translocation of Parkin which primes mitochondria for autophagic elimination. However, whether ROS play a role in the execution of mitophagy is unknown. In the present study, we show that carbonyl cyanide *m*-chlorophenylhydrazone (CCCP) treatment induced both mitochondrial depolarization and generation of ROS that were needed for the mitophagy process. Cells failed to proceed to complete mitophagy if CCCP treatment was discontinued even after recruitment of Parkin and autophagy machinery to mitochondria. Notably, treatment of pro-oxidant was able to replace CCCP treatment to take mitophagy forward, while it alone was insufficient to induce translocation of Parkin to mitochondria or autophagic clearance of mitochondria. In addition, an SOD mimetic that attenuated the superoxide level suppressed mitophagy, while an SOD inhibitor accumulated cellular superoxide and promoted mitophagy. Furthermore, blockage of the p38 signaling pathway inhibited mitophagy induced by ROS, suggesting that it may contribute to the activation of ROS-mediated mitophagy. Together, our study sheds light on the link between ROS and mitophagy at a molecular level, and suggests the therapeutic potential of regulating mitophagy through the superoxide–p38–mitophagy axis.

Reactive oxygen species (ROS )are closely related to human health and diseases through their impact on the signaling transduction pathways and diverse physiological processes. In neurodegenerative diseases, ROS cause damage to proteins, lipids and DNA in neurons.^1, 2^ Gain or loss of function in PD-related genes has been found to increase intracellular ROS levels, thereby promoting ROS-induced cell death.^3, 4^ Autophagy, an important cellular process of quality control, maintains cellular homeostasis by eliminating deleterious damaged proteins or organelles, which would otherwise be accumulated, leading to neurodegenerative diseases.^5, 6^ Although it has been widely considered as a culprit for neurodegenerative diseases, ROS may confer protection to cells by regulating autophagy. For instance, ROS were found to modify a cysteine residue near the catalytic domain of ATG4, thereby activating autophagy.^7^ In addition, Chen *et al.*^8^ reported that superoxide was necessary for starvation-induced autophagy. In contrast, ROS scavengers blocked the formation of autophagosomes and the ensuing degradation of engulfed proteins.^7^

Dysfunction of Parkin/PINK1-dependent mitophagy contributes to the pathogenesis of PD.^9, 10^ In mitophagy, PINK1 phosphorylates and activates Parkin^11, 12^ and ubiquitin.^13, 14, 15^ Phosphorylated ubiquitin further activates Parkin and recruits it to impaired mitochondria.^16, 17^ Subsequently, Parkin ubiquitinates outer mitochondrial membrane (OMM) proteins and triggers their degradation by proteasome,^18, 19^ leading to the clearance of mitochondria via autophagy.^20^ Mitogen-activated protein kinase (MAPK) signaling pathways have been identified to play a role in mitophagy. Slt2, a homolog of mammalian ERK5, and Hog1, a homolog of mammalian p38, function as positive regulators for mitophagy in the yeast *Saccharomyces cerevisiae*.^21^ The dependence of mitophagy on p38 was later replicated in mammalian cells.^22^ Recent studies investigating the effects of ROS on translocation of Parkin to mitochondria have yielded conflicting results. For instance, ROS scavenger was able to inhibit Paraquat-induced Parkin translocation to mitochondria in HeLa cells^20^ and carbonyl cyanide *m*-chlorophenylhydrazone (CCCP)-induced Parkin translocation in mouse embryonic fibroblasts and mouse primary cortical neurons. ^23^ However, ROS scavenger failed to attenuate CCCP-induced Parkin recruitment to mitochondria in HeLa cells.^20^

In contrast to recent focus on the effects of ROS on Parkin dynamics, the role of ROS in execution of mitophagy is less studied. In our present report, we demonstrate that superoxide may represent a key factor that takes mitophagy forward following Parkin recruitment to mitochondria, although it alone has no impact on Parkin dynamics or mitophagy. We further show that the p38 signaling pathway may contribute to the progression of mitophagy induced by ROS. The superoxide–p38–mitophagy axis found in our study may deepen our understanding on the role of ROS in step-wise activation of mitophagy.

## Results

### Recruitment of Parkin and autophagy machinery to mitochondria does not warrant execution of mitophagy

**Figure 1:**
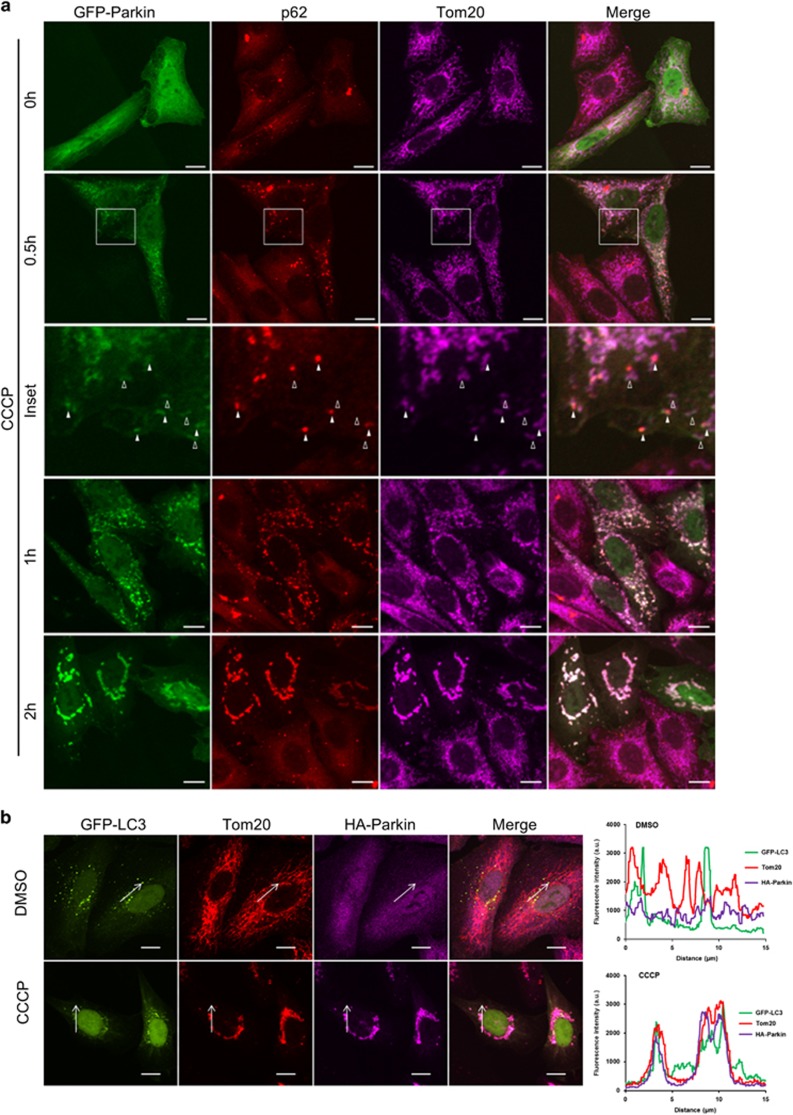

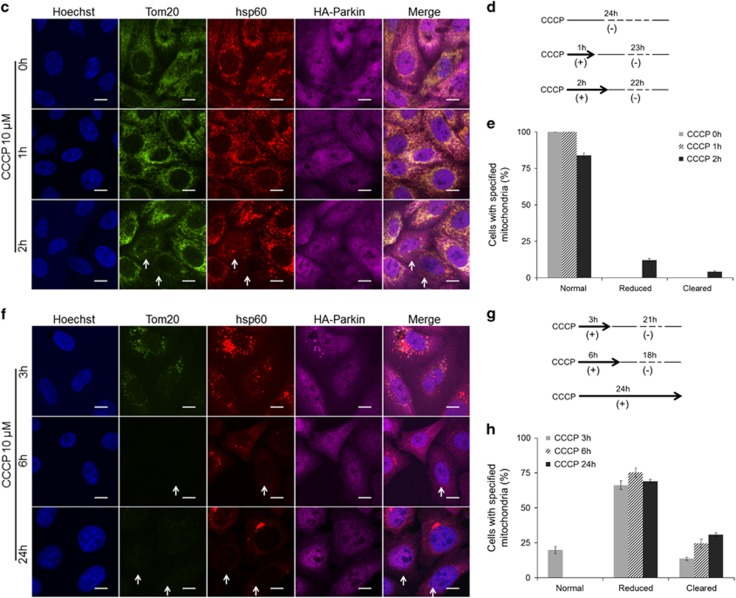
CCCP treatment induces recruitment of Parkin and autophagy component to mitochondria and facilitates execution of mitophagy. (**a**) Representative images of GFP-Parkin expressed HeLa cells treated with CCCP for the indicated times. Cells were stained for p62 (red) and Tom20 (purple). Solid arrowheads indicate mitochondria with both Parkin and p62. Empty arrowheads indicate mitochondria with Parkin but without p62. (**b**) HeLa cells stably expressing HA-Parkin transfected with GFP-LC3 were treated with DMSO or 10 *μ*M CCCP for 2 h. Cells were stained for Tom20 (red) and HA (purple). Line scans next to the images indicate colocalization between LC3 (green) and mitochondria (red) and correlate to the lines drawn in the images. (**c** and **f**) HeLa cells stably expressing HA-Parkin were treated with CCCP as depicted in (**d** and **g**). Cells were stained for Tom20 (green), hsp60 (red) and HA (purple). The arrows in (**c**) indicate representative cells with reduced mitochondrial mass. The arrows in (**f**) indicate representative cells with cleared mitochondrial mass. The average percentages of cells with normal, reduced or cleared mitochondrial mass from (**c**) and (**f**) were presented in (**e**) and (**h**). The error bars represent S.E.M. from three independent experiments; at least 100 cells were analyzed per experiment. Scale bars, 10 *μ*m

Current research on the mechanism of Parkin/PINK1-dependent mitophagy is largely focused on the translocation of Parkin to the damaged mitochondria, with the view that mitophagy will proceed towards completion upon the fulfillment of this prerequisite step. The question we asked was if mitophagy would indeed automatically occur after Parkin recruitment. To address this, we examined mitochondrial translocation of Parkin at different time points post-CCCP treatment. Consistent with previous reports,^24^ Parkin was recruited to the mitochondria as early as 0.5 h post-CCCP treatment and marked overlay of Parkin and mitochondria could be observed after adding CCCP for 1 h (Figure 1a). An autophagy adaptor, p62, was also concurrently stained to monitor its localization. Translocation of p62 to the mitochondria was detected in Parkin-expressing cells also as early as 0.5 h post-CCCP treatment, although p62 was absent in a proportion of Parkin-positive mitochondria (Figure 1a), indicating that p62 translocation to the mitochondria likely lies downstream of Parkin recruitment to mitochondria. We further tested the recruitment of autophagy machinery to the mitochondria using microtubule-associated protein 1A/1B-light chain 3 (LC3), a phagophore/autophagosome marker. Our confocal microscopy data revealed colocalization of LC3 with mitochondria at 2 h post-CCCP treatment (Figure 1b), indicating engulfment of mitochondria by autophagosome. Next, we examined mitochondrial mass in the cells overexpressing Parkin in response to CCCP treatment. CCCP was washed out at the indicated times and mitochondrial mass was determined at 24 h after initial treatment was added (Figures 1c and d). An OMM protein, Tom20, and a mitochondrial matrix protein, hsp60, were stained to visualize mitochondrial proteins at different compartments of the organelle. Mitochondrial mass was assessed using staining of hsp60, as it has been shown that OMM proteins are degraded in a proteasome-dependent manner prior to mitophagy. As shown in Figure 1e, when CCCP was washed out after 2 h of treatment, clearance of mitochondrial mass could only be observed in less than 5% of the cells. When CCCP treatment was extended to 3 h, clearance of mitochondria was greatly increased (Figures 1f–h). Six hours treatment of CCCP had a similar effect on the removal of mitochondria as 24 h of the same treatment (Figures 1f–h). These data indicate that other determinant is necessary for the completion of mitophagy following recruitment of Parkin and autophagy machinery to mitochondria post-CCCP treatment.

### ROS facilitate completion of mitophagy following Parkin translocation to mitochondria

**Figure 2:**
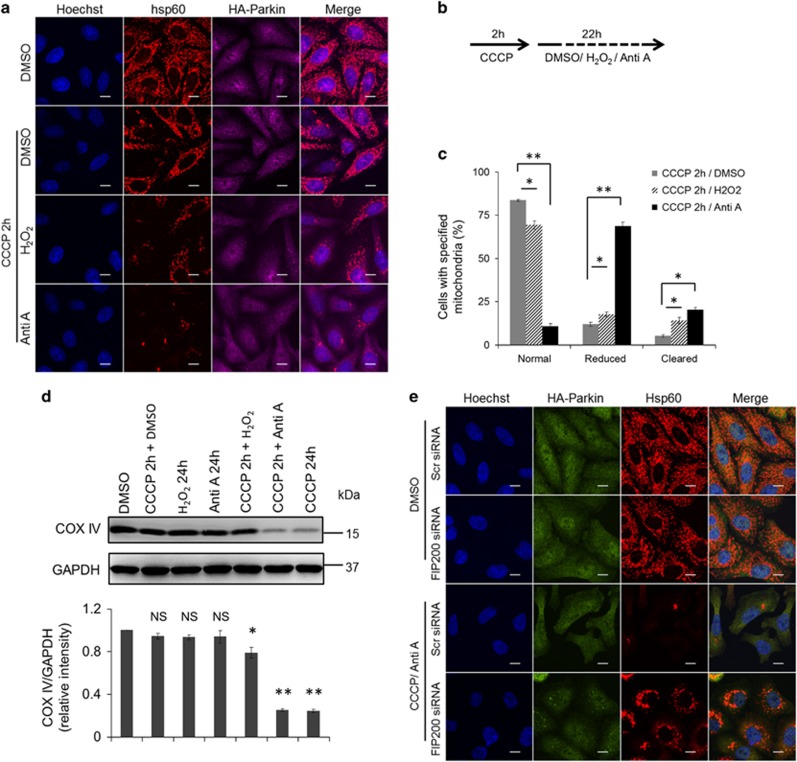
Pro-oxidant is able to push forward autophagic clearance of mitochondria. (**a**) HeLa cells stably expressing HA-Parkin were treated with DMSO, CCCP, antimycin A or H_2_O_2_ as depicted in (**b**). Cells were stained for hsp60 (red) and HA (purple). (**c**) Cells from (**a**) were counted as in Figures 1e and h. The error bars represent S.E.M. from three independent experiments. (**d**) HeLa cells expressing HA-Parkin were treated as indicated and immunoblotted for COX IV and GAPDH. Bottom: Average protein levels of COX IV relative to GAPDH from three independent experiments. (**e**) HeLa cells expressing HA-Parkin were transfected with scramble or FIP200 siRNA. Seventy-two hours post-transfection, cells were treated with DMSO or CCCP/antimycin A as in (**a**) for 24 h. Cells were stained for HA (green) and hsp60 (red). NS, not significant; *P<0.05; **P<0.01 (*t*-test)

**Figure 3:**
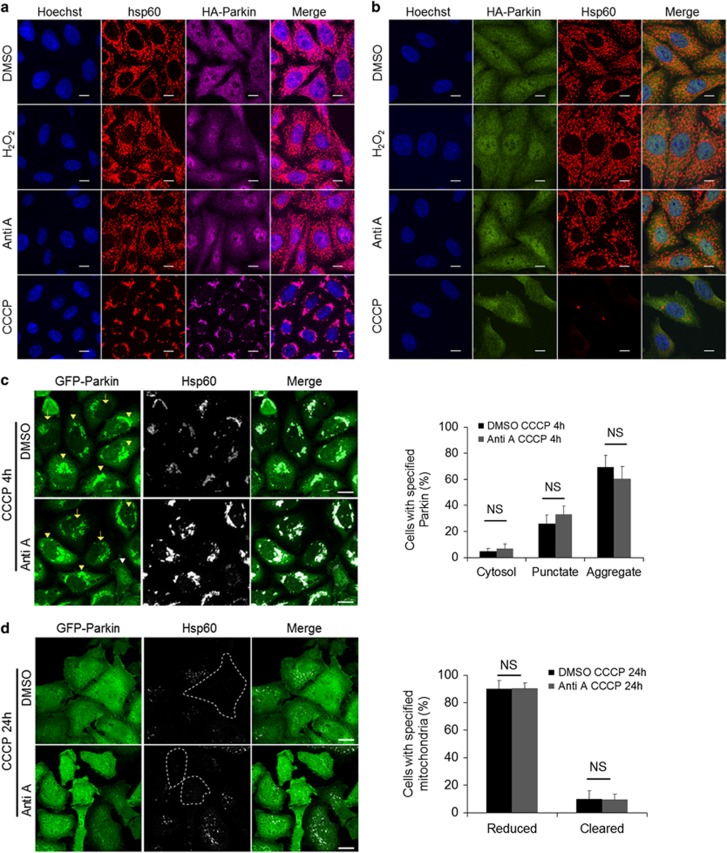
Pro-oxidant alone or its priming has little effect on mitophagy. (**a**) HeLa cells stably expressing HA-Parkin treated with DMSO, H_2_O_2_ (100 *μ*M), antimycin A (2 *μ*M) or CCCP (10 *μ*M) for 2 h. Cells were stained for hsp60 (red) and HA (purple). (**b**) HeLa cells stably expressing HA-Parkin treated with DMSO, H_2_O_2_ (100 *μ*M), antimycin A (2 *μ*M) or CCCP (10 *μ*M) for 24 h. Cells were stained for HA (green) and hsp60 (red). GFP-Parkin-overexpressing cells were treated with DMSO or antimycin A for 2 h prior to CCCP treatment for 4 h (**c**) or 24 h (**d**). Cells were stained with hsp60 (white). Distribution of Parkin and reduction in mitochondrial mass were quantified on the right. White arrowhead indicates cytosolic Parkin; yellow arrows indicate punctate-shaped Parkin; yellow arrowheads indicate Parkin aggregates. NS, not significant

We next sought to determine what factors were involved in the execution of mitophagy. Because CCCP treatment both depolarizes mitochondrial potential and promotes ROS generation, we tested other pro-oxidants to check if mitophagy could be activated by ROS. After being added to the cells for 2 h, CCCP was washed out and replaced with DMSO, antimycin A or H_2_O_2_ for the next 22 h (Figures 2a and b). Indeed, antimycin A or H_2_O_2_ promoted mitochondrial clearance, with antimycin A reducing mitochondrial mass in 70% cells and removing mitochondrial mass in 20% cells (Figures 2a and c). In addition, western blot analysis showed a reduction of COX IV, an inner mitochondrial membrane protein, in the CCCP-pretreated cells that were subsequently exposed to antimycin A or H_2_O_2_ treatment, with antimycin A treatment having a more significant effect (Figure 2d). To further demonstrate if the removal of mitochondria activated by ROS inducer was autophagy-dependent, siRNA targeting FIP200 was used to inhibit autophagy. Our data showed that FIP200 knockdown halted the removal of mitochondria induced by CCCP/antimycin A treatment (Figure 2e), suggesting that this clearance is autophagy-dependent. In order to determine if Parkin recruitment to the mitochondria was a prerequisite for the observed pro-oxidant-mediated effects on mitophagy, Parkin-expressing cells were treated with antimycin A or H_2_O_2_ in the absence of CCCP treatment. In contrast to the observation that CCCP treatment induced robust Parkin translocation to mitochondria, antimycin A or H_2_O_2_ treatment failed to do so (Figure 3a). In addition, mitochondrial mass remained unchanged after 24 h of exposure to antimycin A or H_2_O_2_ in the absence of CCCP pre-treatment (Figure 3b). Furthermore, antimycin A pre-treatment had little effect on CCCP-induced Parkin translocation, aggregation (Figure 3c) and mitochondrial clearance (Figure 3d). Together, our data suggest that ROS promote mitophagy following Parkin translocation to mitochondria.

### Superoxide may facilitate Parkin/PINK1-dependent mitophagy

**Figure 4:**
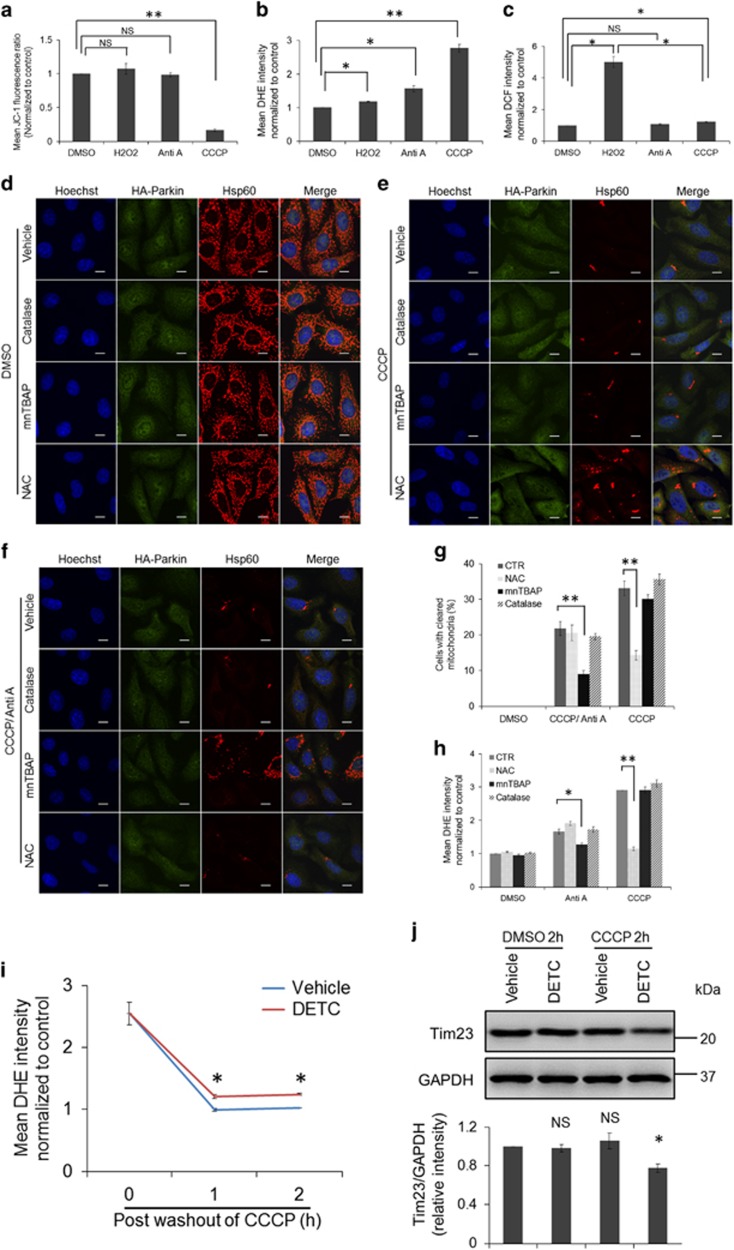
Superoxide promotes the progression of mitophagy. HeLa cells stably expressing HA-Parkin treated with DMSO, H_2_O_2_ (100 *μ*M), antimycin A (2 *μ*M) or CCCP (10 *μ*M) for 2 h. Cells were stained with JC-1 (**a**) or DHE (**b**) and subject to flow cytometric analysis. (**c**) Cells were stained with DCF and washed out prior to treatment of DMSO, H_2_O_2_ (100 *μ*M), antimycin A (2 *μ*M) or CCCP (10 *μ*M) for 1 h. Cells were collected and subjected to flow cytometric analysis. HeLa cells stably expressing HA-Parkin were treated with DMSO (**d**), CCCP for 24 h (**e**) or CCCP for 2 h followed by the treatment of antimycin A for 22 h (**f**) in the presence of vehicle, catalase (1000 U/ml), mnTBAP (200 *μ*M) or NAC (2 mM) for the last 22 h. Cells were stained for HA (green) and hsp60 (red). (**g**) Removal of mitochondrial mass was counted in the cells treated as in (**d**–**f**). (**h**) Cells treated with DMSO, antimycin A or CCCP in the presence of vehicle, catalase (1000 U/ml), mnTBAP (200 *μ*M) or NAC (2 mM) for 2 h were stained with DHE and subject to flow cytometric analysis. (**i**) HeLa cells were treated with CCCP (10 *μ*M) for 2 h prior to the treatment of vehicle or DETC (40 *μ*M) for the indicated times. Cells were stained with DHE and subject to flow cytometric analysis. Comparisons of DHE intensity between the cells treated with DETC and the cells treated with vehicle were performed using *t*-test. (**j**) Cells were treated with CCCP (10 *μ*M) for 2 h prior to the treatment of DMSO or DETC (40 *μ*M) for 22 h. Cells were immunoblotted for Tim23 and GAPDH. Bottom: Average protein levels of Tim23 relative to GAPDH from three independent experiments. NS, not significant; *P<0.05; **P<0.01 (*t*-test)

The major components of ROS include superoxide and hydrogen peroxide, while it was unknown which species was the major regulator for Parkin/PINK1-dependent mitophagy. Before addressing this question, we examined the effects of the drug treatment used in our study on the mitochondria potential. Flow cytometric analysis using JC-1 dye revealed that CCCP treatment triggered significant mitochondrial depolarization as expected (Figure 4a). In contrast, antimycin A or H_2_O_2_ treatment had little effect on mitochondrial potential (Figure 4a). These data suggest that mitochondrial depolarization is not necessary for the execution of mitophagy. Chemical dyes, dihydroethidium (DHE) and 2′,7′-dichlorofluorescin (DCF), were used to indicate intracellular levels of superoxide and hydrogen peroxide, respectively. Flow cytometric data showed that among the drug treatment used in this study, CCCP treatment induced the most significant increase of DHE intensity, followed by antimycin A treatment (Figure 4b). In contrast, either of these two treatment had modest effects on the fluorescence intensity of DCF (Figure 4c). H_2_O_2_ treatment led to marked increase of DCF intensity (Figure 4c) but only stimulated mild increase in DHE intensity (Figure 4b). Together with the data that either CCCP or antimycin A treatment promoted more complete mitophagy than H_2_O_2_ treatment (Figures 2c and d), it is conceivable that execution of mitophagy may be mainly propelled by superoxide. To further examine this notion, ROS scavengers, NAC, mnTBAP and catalase were applied to examine their effects on execution of mitophagy. These ROS scavengers had no effects on mitochondrial mass in control-treated cells (Figure 4d). Interestingly, NAC was only able to halt the progression of mitophagy induced by CCCP treatment (Figures 4e and g), while mnTBAP specifically inhibited execution of mitophagy propelled by antimycin A treatment (Figures 4f and g). Application of catalase had no effect on mitophagy induced either by CCCP or by CCCP/antimycin A treatment (Figures 4e–g). To investigate why these ROS scavengers had distinct effects on CCCP or CCCP/antimycin A-induced mitophagy, DHE fluorescence was monitored upon treatment of the ROS scavengers in the presence of mitophagic inducers. Consistently, catalase, which converts hydrogen peroxide to water had little effect on the fluorescence intensity of DHE. Stimulation of the increase in DHE intensity induced by CCCP treatment was suppressed exclusively by NAC, while increased DHE intensity triggered by antimycin A could only be attenuated by mnTBAP treatment (Figure 4h). It is unknown why NAC and mnTBAP treatment have distinct effects on the generation of superoxide induced by different mitophagic inducers. Notwithstanding this, our data demonstrate that the inhibition of mitophagy by ROS scavengers may be mediated by their effects on superoxide outburst. Furthermore, diethyldithiocarbamate (DETC), an SOD inhibitor, was applied to the CCCP-primed cells. It was found that DETC halted the decrease of DHE intensity subsequent to the washout of CCCP treatment (Figure 4i). Meanwhile, DETC treatment promoted the removal of an inner mitochondrial membrane protein, Tim23, in the CCCP-primed cells (Figure 4j), indicating that the SOD inhibitor promotes mitophagy in the CCCP-primed cells. Together, our data indicate that superoxide may constitute the driving force for the progression of mitophagy.

### p38 signaling pathway contributes to ROS-driven mitophagy

**Figure 5:**
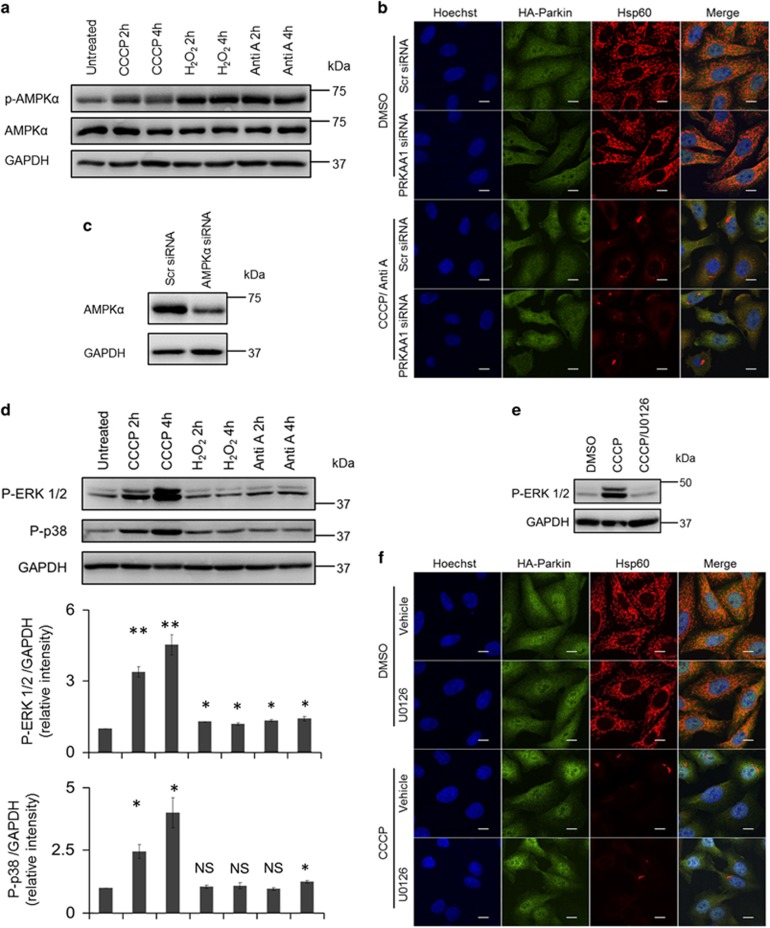
Neither AMPK nor ERK1/2 signaling pathway is responsible for the progression of mitophagy induced by ROS outburst. (**a**) HeLa cells stably expressing HA-Parkin were treated with CCCP (10 *μ*M), H_2_O_2_ (100 *μ*M) or antimycin A (2 *μ*M) for the indicated times. Cells were immunoblotted for phosphorylated AMPK*α*. (**b**) HeLa cells stably expressing HA-Parkin were transfected with scramble or PRKAA1 siRNA. Seventy-two hours post-transfection, cells were treated with DMSO or CCCP/antimycin A as in Figure 2b for 24 h. Cells were stained for HA (green) and hsp60 (red). (**c**) Cells transfected as in (**b**) were immunoblotted for AMPK*α*. (**d**) Cells treated as in (**a**) were immunoblotted for phosphorylated ERK1/2 and p38. Bottom: Average protein levels of phosphorylated ERK1/2 and phosphorylated p38 relative to GAPDH from three independent experiments. (**e**) HeLa cells stably expressing HA-Parkin were treated with DMSO, CCCP (10 *μ*M) in the absence or presence of U0126 for 2 h and subject to western blot analysis for phosphorylated ERK1/2. (**f**) HeLa cells stably expressing HA-Parkin were treated with DMSO or CCCP (10 *μ*M) in the presence or absence of U0126 for 24 h. Cells were stained for HA (green) and hsp60 (red). NS, not significant; *P<0.05; **P<0.01 (*t*-test)

**Figure 6:**
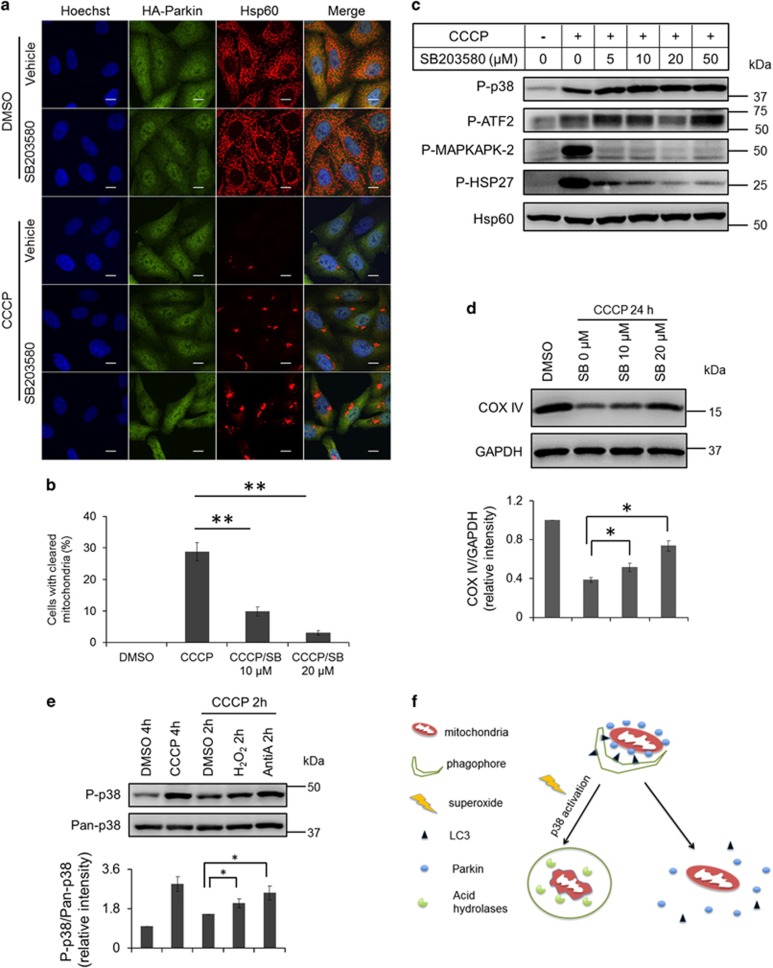
Execution of mitophagy is dependent on p38 signaling. (**a**) HeLa cells stably expressing HA-Parkin were treated with DMSO or CCCP in the absence or presence of 10 *μ*M (fourth row) or 20 *μ*M (fifth row) SB203580 for 24 h and stained for HA (green) and hsp60 (red). (**b**) Cells with cleared mitochondria in (**a**) were quantified. (**c**) HeLa cells stably expressing HA-Parkin were treated with CCCP and SB203580 as indicated, followed by immunoblotting with the indicated antibodies. (**d**) Cells treated with CCCP and SB203580 as indicated were immunoblotted for COX IV and GAPDH. Bottom: Average protein levels of COX IV relative to GAPDH from three independent experiments. (**e**) HeLa cells stably expressing HA-Parkin were treated with DMSO, CCCP (10 *μ*M) for 4 h or CCCP (10 *μ*M) for 2 h followed by the treatment of DMSO, H_2_O_2_ or antimycin A for 2 h. Cells were collected and immunoblotted for endogenous p38 and phosphorylated p38. Bottom: Average protein levels of phosphorylated p38 relative to p38 from three independent experiments. (**f**) A schematic diagram of the role of superoxide-induced p38 activation in the execution of mitophagy. Subsequent to the recruitment of Parkin and LC3 to damaged mitochondria, production of superoxide activates the p38 signaling pathway that is required for the execution of mitophagy, leading to degradation of mitochondria in autolysosome (left path). In the absence of activation of the p38 signaling pathway by superoxide, mitophagy is arrested and Parkin dissociates back to the cytosol (right path). *P<0.05; **P<0.01 (*t*-test).

We next sought to probe the signaling pathway(s) underlying mitophagy propelled by superoxide. It is known that superoxide assumes a major role in the activation of autophagy through the AMPK pathway,^8, 25^ which in turn phosphorylates ULK1^26^ and inhibits mTORC1 via phosphorylation of the mTORC1 subunit raptor.^27^ Since our study showed that superoxide facilitated the execution of mitophagy, we investigated if stimulation of mitophagy by ROS was mediated by the activation of the AMPK pathway. To this end, phosphorylation of AMPK in response to the mitophagic inducers was examined by western blot analysis. As shown in Figure 5a, CCCP treatment did stimulate phosphorylation of AMPK, but to a lesser extent compared with antimycin A or H_2_O_2_ treatment. To further test the necessity of the AMPK pathway in mitophagy, the gene which encodes AMPK catalytic subunit *α*1 (PRKAA1) was silenced using siRNA in Parkin-expressing cells before being exposed to CCCP/antimycin A treatment. The extent of mitochondrial clearance in PRKAA1 knockdown cells was similar to that in control knockdown cells (Figures 5b and c), indicating that the AMPK signaling pathway is not necessary for ROS-mediated mitophagy. Since ERK1/2 and p38 pathways were found to be involved in mitophagy in mammalian cells,^22^ we examined if these two pathways had a role in the mitophagy driven by ROS. Our results showed that CCCP treatment substantially increased phosphorylation of ERK1/2 and p38, while either antimycin A or H_2_O_2_ treatment alone had mild impact on the status of phosphorylation in ERK1/2 and/or p38 (Figure 5d). In addition, U0126, a specific ERK1/2 inhibitor, failed to suppress mitophagy induced by CCCP treatment (Figures 5e and f). Notably, SB203580, a specific inhibitor of p38, was able to suppress the loss of mitochondrial protein hsp60 (Figure 6a). Moreover, 20 *μ*M SB203580 could attenuate mitophagy more potently compared with 10 *μ*M SB203580 (Figures 6a and b). In addition, 20 *μ*M SB203580 had the most significant effects on the phosphorylation of downstream substrates of p38 (Figure 6c). Consistently, western blot analysis showed that SB203580 treatment inhibited elimination of COX IV induced by CCCP treatment (Figure 6d). We further monitored the phosphorylation of p38 in response to antimycin A or H_2_O_2_ treatment in the presence of CCCP pre-treatment. Although antimycin A alone only slightly increased phosphorylation of p38 (Figure 5d), antimycin A or H_2_O_2_ treatment retained the phosphorylation of p38 stimulated by CCCP treatment, with antimycin A treatment having a more considerable effect than H_2_O_2_ treatment (Figure 6e). Consistently, antimycin A or H_2_O_2_ treatment could only induce mitophagy in the presence of CCCP pre-treatment (Figures 2a, c, d and 3b). Together, our results suggest that the activation of the p38 signaling pathway may be involved in ROS-driven mitophagy.

## Discussion

The mechanism of mitophagy is continually being unravelled, especially the process underlying Parkin recruitment to damaged mitochondria. However, how subsequent mitophagy proceeds may be more complicated than expected. The repertoire of components utilized in mitophagy may be very unique, though some of them are shared by both mitophagy and general autophagy. For instance, in mitophagy, the transcription factor EB (TFEB), a regulator of lysosomal biogenesis, is activated in a manner different from starvation-induced autophagy.^28^ The present study is, to our knowledge, the first attempt to investigate the driving force for mitophagy following Parkin translocation to mitochondria. Mitophagy was halted following the withdrawal of CCCP treatment, even when Parkin, p62 and LC3 had translocated to mitochondria. Treatment of pro-oxidant, including antimycin A and H_2_O_2_, was able to propel mitophagy in the absence of further CCCP treatment, suggesting that ROS may contribute to the execution of mitophagy. Our data also place the role of ROS downstream of Parkin redistribution to mitochondria in the process of mitophagy, as antimycin A or H_2_O_2_ was able to facilitate clearance of mitochondria after Parkin translocation to mitochondria but failed to do so if the translocation was not triggered beforehand. In addition, our results suggest that superoxide may be the major species of ROS that propels mitophagy. Supporting this, we show that the regulation of superoxide by SOD mimetic or inhibitor was able to interfere with the progression of Parkin/PINK1-mediated mitophagy.

Moreover, the p38 signaling pathway may constitute the molecular cascade linking ROS and mitophagy. In our study, inhibition of p38 substantially suppressed mitophagy despite the stimulation of ROS generation by CCCP treatment, indicating that ROS promote mitophagy through the activation of p38 pathway. In other words, p38 is a key downstream effector of ROS needed for the completion of the mitophagy event. Thus, our data and previous study demonstrate that the p38 pathway may represent a conservative signaling pathway regulating mitophagy in yeast^21^ and human, induced by starvation, hypoxia^22^ or ROS. In contrast, ERK1/2 may be dispensable in the Parkin/PINK1-dependent mitophagy under the present experimental conditions, although it is necessary for Parkin-independent mitophagy induced by starvation.^22^ In addition, ROS-induced AMPK activation has been reported to mediate starvation-induced autophagy, through its inhibition of mTOR.^25^ However, this cellular event lies upstream of the process to initiate autophagy.^29^ In contrast, driving mitophagy forward by superoxide in our study is further down in the downstream cascade, as it was required even after autophagosome maker, LC3, had translocated to the mitochondria. Thus, as shown in Figure 6f, we propose that superoxide act downstream of Parkin translocation to mitochondria to drive mitophagy forward, while activation of p38 signaling pathway may contribute to the completion of mitophagy. Identification of the driving force of mitophagy may have physiological significance, as therapeutic strategies may be developed to regulate mitophagy by targeting superoxide and/or p38.

## Materials and Methods

### Plasmid construction and transfection

HA-Parkin and GFP-LC3 were obtained from Addgene (Cambridge, MA, USA) (Plasmid 38248 and 21073). Full-length Parkin cDNA was subcloned into C1 vector (Addgene plasmid 54607) to generate GFP-tagged Parkin construct. Scrambled siRNA (ON-TARGETplus Non-targeting Pool, D-001810-10-05; Dharmacon, Lafayette, CO, USA), FIP200 siRNA (SI02664578; Qiagen, Venlo, Netherlands) and PRKAA1 siRNA (SIHK1776; Sigma, St. Louis, MO, USA) were used in our study. siRNAs were transfected into HeLa cells using Lipofectamine RNAiMAX Transfection Reagent (Thermo Fisher Scientific, Waltham, MA, USA). Seventy-two hours post-transfection, cells were treated as indicated.

### Cell culture and treatment

HeLa cell line (ATCC, Manassas, VA, USA) was maintained in Dulbecco’s modified Eagle's medium media with 10% fetal bovine serum and 1% penicillin/streptomycin at 37 °C under 5% CO_2_ conditions. Stable HA-Parkin HeLa cell line was generated as described previously.^30^ CCCP, antimycin A, catalase, NAC and sodium diethyldithiocarbamate trihydrate were purchased from Sigma. SB203580 was purchased from Synkinase (Parkville, VIC, AU). U0126 was purchased from Cell Signaling (Danvers, MA, USA). mnTBAP and H_2_O_2_ were purchased from Merck (Billerica, MA, USA). To stimulate Parkin translocation to mitochondria, Parkin-overexpressing cells were incubated with 10 *μ*M CCCP for 2 h. To induce mitophagy, Parkin-overexpressing cells were treated as indicated.

### Immunofluorescence

For immunostaining, cells were fixed with 4% paraformaldehyde in PBS, permeabilized and blocked with PBS containing 0.5% Triton X-100 and 3% BSA for 30 min at room temperature. The primary antibodies used were mouse anti-HA (sc-7392; Santa Cruz Biotechnology, Dallas, TX, USA, 1:200 dilution), mouse anti-p62 (ab56416; Abcam, Cambridge, UK, 1:500 dilution), rabbit anti-Tom20 (sc-11415; Santa Cruz Biotechnology, 1:200 dilution) and goat anti-hsp60 (sc-1052; Santa Cruz Biotechnology, 1:200 dilution). The secondary antibodies used were Alexa Fluor dye-conjugated donkey anti-mouse, donkey anti-goat and donkey anti-rabbit (Thermo Scientific, Waltham, MA, USA 1:200 dilution). Nuclei were counterstained with Hoechst 33342 (Thermo Scientific). All fluorescent images were acquired on Yokogawa CSU (confocal scanner unit)-W1 spinning disc system (Andor, Belfast, UK) equipped with a 60 × 1.49 NA objective (Nikon, Tokyo, Japan). The brightness of the images was adjusted using NIS-Elements (Nikon).

### Immunoblotting

The cells were solubilized in M-PER reagent (Thermo Scientific) supplemented with protease inhibitors. The lysates were clarified by centrifugation at 16 000 × *g* for 30 min at 4 °C. Protein samples were loaded and separated on 6, 10 or 12% SDS-PAGE gel and immunoblotted with antibodies including mouse anti-GAPDH (sc-47724; Santa Cruz Biotechnology, 1:1000 dilution), mouse anti-Tim23 (611222; BD Biosciences, San Jose, CA, USA, 1:1000 dilution) and rabbit anti-hsp60 (12165; Cell Signalling, 1:1,000 dilution), rabbit anti-COX IV (4850; Cell Signalling, 1:2500 dilution), rabbit anti-AMPK*α* (5832; Cell Signalling, 1:1000 dilution), rabbit anti-phospho-AMPK*α* (2535; Cell Signalling, 1:1000 dilution), rabbit anti-p38 (8690; Cell Signalling, 1:1000 dilution), rabbit anti-phospho-p38 (4511; Cell Signalling, 1:1000 dilution), rabbit anti-phospho-ERK1/2 (4370; Cell Signalling, 1:1000 dilution), rabbit anti-phospho-ATF-2 (5112; Cell Signalling, 1:1000 dilution), rabbit anti-phospho-hsp27 (9709; Cell Signalling, 1:1000 dilution) and rabbit anti-phospho-MAPKAPK-2 (3007; Cell Signalling, 1:1000 dilution).

### ROS and mitochondrial potential detection

To test levels of ROS and mitochondrial potential, fluorescent indicator DHE (Invitrogen), DCF (Sigma) and JC-1 (Invitrogen) were used. Cells were harvested, washed in PBS and stained with 5 *μ*M DHE or 2 *μ*M JC-1 dyes for 30 min at 37 °C in the dark. For DCF detection, cells were stained with the dye for 30 min at 37 °C and washed out at 1 h prior to analysis. Fluorescence was measured immediately with LSRFortessa (BD Biosciences), and the data were analyzed with the FACSDiva version 6.2 software (BD Biosciences). The fluorescence intensity of the red signal in DHE-stained cells and green signal in DCF-stained cells indicate the levels of superoxide and hydrogen oxide, respectively. The ratio of the red to green fluorescence intensity in JC-1-stained cells was used to represent mitochondrial membrane potential.
