# Correction: Superoxide drives progression of Parkin/PINK1-dependent mitophagy following translocation of Parkin to mitochondria

**DOI:** 10.1038/s41419-018-0832-2

**Published:** 2018-07-19

**Authors:** Bin Xiao, Xiao Deng, Grace GY Lim, Shaoping Xie, Zhi Dong Zhou, Kah-Leong Lim, Eng-King Tan

**Affiliations:** 1 Department of Neurology, National Neuroscience Institute, Singapore, Singapore; 2 Neurodegeneration Research Laboratory, National Neuroscience Institute, Singapore, Singapore; 3 Department of Research, National Neuroscience Institute, Singapore, Singapore; 4 Department of Physiology, National University of Singapore, Singapore, Singapore; 5 Department of Neurology, Singapore General Hospital, Singapore, Singapore; 6 Duke-NUS Medical School, National University of Singapore, Singapore, Singapore

**Correction to**: *Cell Death & Disease*; 10.1038/cddis.2017.463; published online 12 October 2017.

The PDF and HTML versions of the article have been updated to include the Creative Commons Attribution 4.0 International License information.

